# Clinical presentation, radiographic findings, and treatment outcomes in children with adenoid hypertrophy in a paediatric outpatient clinic in Enugu, Nigeria

**DOI:** 10.4314/gmj.v57i3.7

**Published:** 2023-09

**Authors:** Ijeoma O Ohuche, Nneka I Iloanusi, Chinedu M Dike, Ethel N Chime

**Affiliations:** 1 Department of Paediatrics, University of Nigeria Teaching Hospital, Ituku-Ozalla, Enugu; 2 Department of Paediatrics, Niger Foundation Hospital and Diagnostic Centre, Enugu; 3 Department of Radiation Medicine, Faculty of Medical Sciences, College of Medicine, University of Nigeria, Ituku-Ozalla Campus, Enugu; 4 Department of Family Medicine, Niger Foundation Hospital and Diagnostic Centre, Enugu; 5 Department of Otorhinolaryngology, Faculty of Medical Sciences, College of Medicine, University of Nigeria, Ituku-Ozalla Campus, Enugu

**Keywords:** adenoidal hypertrophy, postnasal space, Paediatrics, Nigeria

## Abstract

**Objectives:**

To determine the clinical presentation, imaging features and outcomes of children with adenoid hypertrophy in our setting.

**Design:**

A retrospective study.

**Setting:**

The paediatric clinic of a private hospital in Enugu.

**Participants:**

51 children, aged 2 to 108 months, with suggestive clinical features and radiographic report of adenoid hypertrophy who presented over 3 years

**Interventions:**

Clinical information was obtained from the patient's medical records. Data was analysed for the clinical characteristics of the patients, the relationship between the degree of airway narrowing on a postnasal space (PNS) radiograph and treatment outcomes.

**Main outcome measures:**

Degree of airway narrowing as measured on a PNS radiograph, the type of and outcomes of treatment

**Results:**

There was an almost equal male (54.7%): female (45.1%) ratio in the occurrence of adenoid hypertrophy, with a mean age of occurrence of 31.50 ± 3.64 months. Noisy breathing was the commonest symptom (94.1%); history of atopic rhinitis in 64.7% of cases and hyperactive airway disease in 45.1% more than 50% of cases with airway narrowing resolved with medical management only.

**Conclusion:**

Adenoid hypertrophy should be considered in evaluating the upper airway in children under five. Paediatricians should be conversant with diagnosing and managing this common cause of upper airway obstruction.

**Funding:**

None declared

## Introduction

The adenoids are a mass of lymphoid tissue located in the roof and posterior wall of the nasopharynx.^1^ Together with the tonsils, they form part of the Waldeyer ring, a ring of lymphoid tissue in the pharynx which constitutes part of the body's defence against respiratory pathogens.^2^ Following infection of the upper respiratory tract, multiplication of lymphoid follicles of the adenoids leads to hyperplasia and hypertrophy of the adenoids.^3^ In addition to upper respiratory tract infections, allergies may also result in enlargement of the adenoids.^4^ Hypertrophy of the adenoids is an important phenomenon since hypertrophic adenoids obstruct the posterior choanae, impeding nasal airflow and drainage of secretions and leading to various clinical manifestations.

Adenoid hypertrophy is a common condition in children^5^, with an estimated prevalence of 19-58% in children between 6 months and 15 years of age.^6^ It is a leading cause of upper airway obstruction and nasal airflow limitation in the paediatric population.^7^ In addition to obstructive symptoms of the upper airway, including obstructive sleep apnoea, hypertrophy of the adenoids is associated with other complications in children, such as chronic sinusitis, recurrent otitis media with effusion, chronic serous otitis media, pulmonary hypertension, cor pulmonale, craniofacial anomalies and failure to thrive.^8^ This condition is more common in children than adults since the adenoids naturally involute with age.^9^ Some studies have demonstrated an association between adenoid hypertrophy and allergies in children.^10^

Earlier studies have been carried out in our locality on the prevalence of adenoid hypertrophy among children. The current study aimed at documenting a more recent analysis of the pattern of presentation, as well as clinical correlates of adenoid hypertrophy among children in our environment, especially in the wake of a rapid rise of allergic disorders worldwide.^11^

## Methods

This study was carried out at the paediatric outpatient clinic of a private hospital in Enugu. The hospital caters to patients who reside in Enugu and its environs, and the children who attend the hospital's paediatric clinic reside in the Enugu metropolis mainly (a civil-service dominated town) and belong to an average or above-average socio-economic class. The hospital's Research Ethics Committee granted approval for this study (Number: NFH-IRB 2022/0003) to access retrospective data from patients' folders. The study sought to document the clinical characteristics, radiographic features and treatment outcomes of identified patients with adenoid hypertrophy among children attending the paediatric outpatient clinic of the hospital over three years (from July 2018 - June 2021).

Data was obtained from the hospital register and the patient's case notes. First, paediatric patients with a preliminary diagnosis of adenoid hypertrophy, based on the clinical history and examination findings, were identified from the hospital register. The initial diagnosis was then confirmed by the report of a lateral radiograph of the neck and postnasal space, which was obtained from the patients' folders.

**Figure 1:**
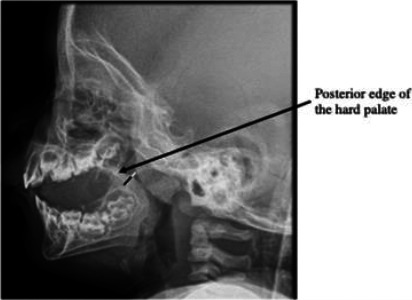
Lateral radiograph of the postnasal space showing Cohen's method for assessing adenoidal enlargement. The thickness of the soft palate (thick black line) is measured 1cm away (0.5cm in children < 3 years of age) from the posterior edge of the hard palate (black arrow). The width of the adjacent nasopharyngeal airway (short white line) is also measured. In this child, the airway is narrower than half the thickness of the soft palate; the adenoid (lobulated soft tissue density at the posterior pharyngeal wall) was therefore reported as severely enlarged

All patients under 18 years, with a radiographic report of adenoid hypertrophy, who presented to the hospital within the study timeframe were included (total population sampling). Data regarding demographic characteristics, clinical presentation, and treatment outcomes of patients who met the inclusion criteria was obtained from their case notes. In the three years studied, 61 children presented with symptoms suggestive of adenoid hypertrophy. However, ten of these had missing data x-ray reports and were excluded from the study; therefore, fifty-one children were eligible for the study. DICOM images of the postnasal X-rays were available for further assessment in 34 out of the 51 children studied. All radiographs were taken with a Computed Radiography (CR) unit (Italray Statix X-ray machine with Carestream Classic CR Digitizer system). With the restrained child lying decubitus on the x-ray couch, standard protocols were observed- a film/focus distance of 2 meters, with radiographic factors of 60mAs and 7KV. The incident ray was centred 5cm behind the nasion. The radiologist's assessment of adenoidal hypertrophy was based on the method proposed by Cohen and Konak.^12^ It is a simple and reproducible method of assessing the degree of adenoidal enlargement by measuring the thickness of the soft palate, 1cm below the posterior limit of the hard plate in children 3 years and above (0.5cm in children below 3 years) and comparing it to the width of the adjacent nasopharyngeal airway. If the airway is wider than the thickness of the soft palate, the adenoid is reported as mildly enlarged. If the airway is narrower than the thickness of the soft palate, the adenoid is reported as moderately enlarged. If the airway is narrower than half the thickness of the soft palate, the adenoid is reported as severely enlarged (Figure 1).

The procedure for adenoidectomy was as follows: with the patient in the supine position and the neck extended, the oropharynx was exposed with the Boyle Davis mouth gag. The gag was then anchored on a bipod stand and stabilised on the Mcqauran plate. The palate was then palpated for the submucous cleft, and an oropharyngeal pack was put appropriately with Magill forceps. The uvula and soft palate were retracted with the pillar retractor and held with the left hand, and the adenoid tissue could be viewed and/or palpated with the right hand.

The Laforce adenotome was held like a pen in the right hand, gently introduced into the oropharynx, and rotated so that the basket of the adenotome was insinuated towards the nasopharynx. With the blade open, the basket engaged the adenoid tissue and pressed it against the nasopharyngeal wall. The blade was then closed, and the adenoid was removed with a downward sweep-like motion. The lateral bands were removed using the adenoid curette, and the adenoid bed was palpated or viewed to ensure no remnant tissue.

## Results

Fifty-one children were studied, all with a radiological report of adenoid hypertrophy. These were aged 2 to 108 months, with a mean age of 31.50 ± 3.64 months. Most of the study population (86.7%) were under 60 months of age. Of the total population, 54.9% were male, while 45.1% were female. More than half (62.7%) of the participants had access to health insurance (Table 1).

**Table 1:** Socio-demographic and mode of payment characteristics of the study participants

Variable	Frequency n(%)	Mean +/- SD
**Age of study participants (in months)**	33 (64.7)	31.50 ± 3.64 (Range: 24.19-38.80)
**Gender**		
**Male**	28 (54.9)	
**Female**	23 (45.1)	
**Mode of payment for healthcare services**		
**Out-of-pocket**	19 (37.3)	
**Insurance**	32 (62.7)	

**Figure 2:**
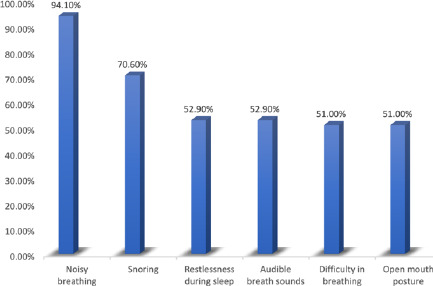
Various clinical presentations of the study participants

Noisy breathing accounted for the most common symptom at presentation, seen in 94.1% of the study population, and this was followed by snoring (70.6%). Other symptoms, such as difficulty in breathing, mouth-breathing and restlessness during sleep, were seen in more than half of the patients. An open-mouth posture and audible breath sounds were found on the physical examination of the participants in more than 50% of cases (Figure 2).

Nutritional complications of adenoid hypertrophy, such as feeding difficulties and poor weight gain, were present in a minority of cases (2% for each complication). However, other complications, such as recurrent ear discharge (5.9%) and recurrent tonsillitis (9.8%), were more commonly observed. A high prevalence of atopy was observed in the study population, with a 64.7% prevalence of atopic rhinitis and a 45.1% prevalence of hyperactive airway disease among study participants. (Table 2).

**Table 2:** Associations and complications of adenoid hypertrophy among the study population

Associations	Frequency (%)
**Atopic rhinitis**	33 (64.7)
**Hyperactive airway disease**	23 (45.1)
**Recurrent tonsillitis**	5 (9.8)
**Otologic symptoms**	3 (5.9)
**Complications**	
**Feeding difficulties**	4 (7.8)
**Poor weight gain**	1 (2.0)

Medical management was successful in 56.9% of the study population, while 15.7% required surgical management (adenoidectomy). Approximately one-quarter (23.5%) of patients were lost to follow-up after commencement of medical treatment. Recurrence of symptoms was observed in a few cases, following medical management (2.0%), as well as surgical management (2.0%) (Table 3).

**Table 3:** Relationship between degree of adenoid enlargement on X-ray and treatment outcomes of the patients

Degree of adenoid enlargement on X-ray	Mild N (%)	Moderate N (%)	Severe N (%)	Total N (%)
**Resolution with medical management**	5 (55.6)	12 (66.7)	5 (71.4)	22 (64.7)
**Surgical management**	3 (33.3)	4 (22.2)	1 (14.3)	8 (23.5)
**Recurrence after surgery**	0	0	1(14.3)	1 (3.0)
**Recurrence after medical management**	0	1 (5.6)	0	1 (3.0)
**Lost to follow up**	1 (11.1)	1(5.6)	0	2 (5.9)
**Total**	9 (100)	18 (100.1)	7 (100)	34 (100.1)

## Discussion

This study reported 51 children with upper airway obstruction secondary to adenoid hypertrophy presenting to our paediatric outpatient clinic over three years. The figure is about twice the number of cases documented by Chinawa et al. in 2015 in a similar locality.^13^ This finding suggests a rise in the incidence of adenoid hypertrophy among children in our locality, the cause of which may not be easily deduced. One possible explanation may be the rising incidence of allergies noted worldwide,^11^,^14^ since some association has been found between adenoid growth and regrowth and the presence of allergies in children.^10^,^15^,^16^ A possible mechanism of this association is that of hyperplasia and hypertrophy of lymphoid tissues seen in allergies, which leads to an increase in the size of the tonsils and adenoids (lymphoid tissues), and which may result in symptoms of upper airway obstruction.^15^ Notably, it has also been documented that adequate treatment for allergic rhinitis may lead to a reduction in the occurrence of adenoid hypertrophy in children with allergies.^17^

The substantial number of children with adenoid hypertrophy in our clinic sharply contrasts with conclusions from other studies,^13^ in which adenoid hypertrophy was commoner in children from lower socio-economic backgrounds since all our patients belonged to middle-class or above-average families. This is in keeping with the epidemiology of allergic disorders (posited to be associated with adenoid growth and hypertrophy), which are commoner among children from higher-income families.^14^ This positive association between allergies and adenoid hypertrophy was seen in our study population since approximately two-thirds of our patients had a history suggestive of atopy. Similar results have been documented by other studies, such as that of Olusesi et al.,^18^ who found a relationship between the presence of allergic rhinitis and an early onset of adenotonsillar disease among Nigerian children; and Sadeghi-Shabestari et al.,^19^ who concluded that allergy and sensitivity to allergens are important risk factors for the development of adenotonsillar hypertrophy in children.

Approximately three-quarters of our study population was aged 2-36 months, with a mean age at presentation of 31 ± 26.15 months and a peak age at presentation of 36 months. This finding is in keeping with the physiology and natural history of the adenoids, which have been shown to grow from birth till about twelve years of age; the greatest increase in size occurs between the ages of two and eight years, with a peak at five years of age. Thereafter, the adenoids begin to involute and atrophy from about eight to ten years of age. Complete atrophy is seen around adolescence, and this lymphoid tissue is hardly seen in adults.^2^,^20^ A higher prevalence of adenoid hypertrophy is expected in the years before the onset of regression of the adenoids, as seen in our study population. The oldest patient in this study was aged 108 months (nine years of age) at presentation, and there was no report of this disease condition in children aged ten years or above in our study- a finding which is also in keeping with the natural history of the adenoids. Unexpectedly, however, 27.5% of our cases were aged below 12 months of age. This is surprising since adenoid hypertrophy is generally uncommon in infants.^21^ There is some documentation that though the adenoids become visible from three to six months of age, pathological encroachment of the nasopharynx by the adenoids does not occur until about 1 to 2 years.^17^ Our findings showed that more than one-quarter of cases of adenoid hypertrophy in our study population occurred in children aged less than one year. Cases of severe upper airway obstruction secondary to adenoid hypertrophy in infants and even neonates have been described in the literature.^23^,^24^ Thus, adenoid hypertrophy should not be excluded as a possible aetiology of upper airway obstruction in children who are yet to celebrate their first birthday.

Adenoid hypertrophy is an obstructive condition with different presenting features depending on the area obstructed.^25^ Symptoms of nasal obstruction were the commonest presenting feature in our patients, who presented mainly with noisy breathing (94.1%), mouth-breathing (70.6%) and sleep-disordered breathing (70.6%). Other features of nasal obstruction, such as an open-mouth posture and audible breath sounds on presentation, were seen in more than 50% of our patients. Ninety-eight per cent of patients presented with more than one symptom of upper airway obstruction. Obstruction of the Eustachian tube was uncommon in our study, as only 5.9% of our patients presented with otalgic symptoms. Adenoid hypertrophy should, therefore, be considered in assessing children with features of upper airway obstruction, especially in the presence of co-existent atopy.

Surgery has remained the mainstay of management of children with moderate to severe symptomatic adenoid hypertrophy, especially with persistent recurrence of obstructive symptoms or recurrent infection.^26^ Most commonly, excision through the mouth is done using an adenoid curette.^27^

However, some studies have demonstrated the efficacy of intranasal steroids (INS) in managing adenoid hypertrophy, especially with prolonged daily medication use. INS have been shown to mediate their effects by suppressing inflammation and altering the adenoid bacterial flora, thereby reducing the risk of adenoid infection.^28^ The efficacy of INS in managing adenoid hypertrophy has been documented by some randomised control trials, which have shown an up to five-fold reduction in adenoid size following daily use of INS.^28^ This study's findings agree with these outcomes, as 56.9% of our population responded to medical management using daily INS (fluticasone furoate) for an average of two weeks. In addition, patients also received oral antibiotics as well as antihistamines. However, approximately 15% of our patients required surgical management because of the severity of their symptoms or following ineffectiveness of INS. Literature shows that full recovery following adenoidectomy is usually the rule in children, who usually experience minimal discomfort following surgery. This was the case in our study, in which adenoidectomy was well tolerated by 15.7% of patients who required surgical management. There were no documented post-operative complications.

The study showed that the majority of patients who presented with mild, moderate or severe adenoid enlargement on X-ray of the postnasal space responded to medical management and therefore, the degree of airway obstruction on X-ray was not a sufficient predictor of response to medications or need for surgical management. While 33.3% of patients with mild airway obstruction on X-ray required surgical management, only 14.3% of patients with severe obstruction required surgery. These findings corroborate data from other studies, such as those of Yaseen et al.,^29^ and Lourenço et al.,^30^ which showed that nasopharyngolaryngoscopy was a much more reliable method of assessing the adenoid size and degree of a nasopharyngeal obstruction than a lateral x-ray of the neck. They, therefore, concluded that the size of the adenoids intraoperatively, as well as the degree of airway obstruction, were more closely related to findings on nasendoscopy than those of radiologic investigations. Other authors have found that lateral neck X-rays remain a non-invasive, relatively cheap, readily available and accurate modality for assessing adenoid size and degree of nasopharyngeal obstruction.^31^,^32^ In a setting such as ours where nasal endoscopy is not readily available, and when available, may not be easily afforded, a lateral neck X-ray may remain the investigation of choice in evaluating children with adenoid hypertrophy. However, as seen from our study, the decision for surgical management should not depend solely on the degree of airway obstruction seen on X-ray since a great majority of children with severe X-ray findings may respond to medical management of the disease condition.

## Conclusion

Adenoid hypertrophy is a common cause of airway obstruction in the first five years of life, with a peak incidence between 1 and 3 years of age. The size of the adenoids or degree of airway obstruction seen on lateral postnasal-sp radiographs are inadequate predictors of treatment outcomes. Response to medical management was excellent in more than fifty per cent of cases, and surgical removal of the adenoids is well tolerated in the paediatric population when carried out by skilled otorhinolaryngologists. Paediatricians should be very conversant with diagnosing and managing this common cause of upper airway obstruction in under-fives.
